# TaO_*x*_-based resistive switching memories: prospective and challenges

**DOI:** 10.1186/1556-276X-8-418

**Published:** 2013-10-09

**Authors:** Amit Prakash, Debanjan Jana, Siddheswar Maikap

**Affiliations:** 1 Thin Film Nano Technology Laboratory, Department of Electronic Engineering, Chang Gung University, Tao-Yuan 333, Taiwan

**Keywords:** Resistive switching, Memory, TaO_*x*_, RRAM

## Abstract

Resistive switching memories (RRAMs) are attractive for replacement of conventional flash in the future. Although different switching materials have been reported; however, low-current operated devices (<100 μA) are necessary for productive RRAM applications. Therefore, TaO_*x*_ is one of the prospective switching materials because of two stable phases of TaO_2_ and Ta_2_O_5_, which can also control the stable low- and high-resistance states. Long program/erase endurance and data retention at high temperature under low-current operation are also reported in published literature. So far, bilayered TaO_*x*_ with inert electrodes (Pt and/or Ir) or single layer TaO_*x*_ with semi-reactive electrodes (W and Ti/W or Ta/Pt) is proposed for real RRAM applications. It is found that the memory characteristics at current compliance (CC) of 80 μA is acceptable for real application; however, data are becoming worst at CC of 10 μA. Therefore, it is very challenging to reduce the operation current (few microampere) of the RRAM devices. This study investigates the switching mode, mechanism, and performance of low-current operated TaO_*x*_-based devices as compared to other RRAM devices. This topical review will not only help for application of TaO_*x*_-based nanoscale RRAM devices but also encourage researcher to overcome the challenges in the future production.

## Review

### Background

**Figure 1:**
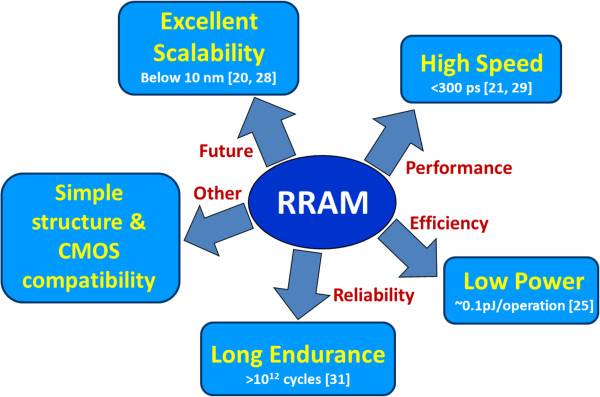
**Prospective of RRAM devices.** Endurance, speed, scalability, and requirements of RRAM devices.

Semiconductor memory is an essential component of today’s electronic systems. It is used in any equipment that uses a processor such as computers, smart phones, tablets, digital cameras, entertainment devices, global positioning systems, automotive systems, etc. Memories constituted 20% of the semiconductor market for the last 30 years and are expected to increase in the coming years [1]. Generally, memory devices can be categorized as 'volatile’ and 'non-volatile’ based on their operational principles. A volatile memory cannot retain stored data without the external power whereas a non-volatile memory (NVM) is the one which can retain the stored information irrespective of the external power. Static random access memory and dynamic random access memory (DRAM) fall into the volatile category, while 'Flash’ which is the short form of 'flash electrically erasable programmable read-only memory’ is the dominant commercial NVM technology. The requirements of an ideal NVM are high density, scalability, low cost, low-energy operation, and high performance for potential applications. Today’s dominant memory technologies are DRAM and Flash, both have scaling issues. The DRAM offers very high endurance (approximately 10^14^ cycles); however, the endurance of Flash is limited (approximately 10^6^ cycles), and the operation is slow as the program/erase time is relatively high (microseconds up to milliseconds). Generally, it needs high voltage for program and erase operations (>׀10 ׀V) [2,3]. In order to overcome these problems, other non-volatile memories such as ferroelectric RAM (FeRAM) [4,5], magnetic RAM (MRAM) [6,7], phase-change-memory (PCM) [8], and resistive RAM (RRAM) are being investigated [9-25]. The basic memories, prototypical, and emerging memories with respect to various performance parameters from International Technology Roadmap for Semiconductors (ITRS) in 2012 have been compared [26]. All these memories store data by resistance change in contrast to charge as in basic memories. In FeRAM, the polarization direction of the dipoles in the ferroelectric layer can be switched by applying the electric field which, in turn, leads the different memory states. MRAM utilizes the orientation of magnetization of a small magnetic element by the application of magnetic field which gives rise to the change in the electric resistance and enable data bits to be stored. Although, FeRAM and MRAM both have fast switching (<20 ns) and long endurance (>10^15^ cycles), these memories show insufficient scalability [27]. Moreover, MRAM needs high programming current (in the range of milliampere) [6]. Compared to FeRAM and MRAM, PCM offers greater potential for future application because of its better scalability [27]. In principle, PCM heats up a material changing it from low-resistance polycrystalline phase to a high-resistance amorphous phase reversibly. So in PCM, the generated heat, i.e., thermal effect, controls the switching. Due to this, the PCM cell needs more power for switching which limits its application in low-power devices. All memories discussed above are in production, though RRAM is at its early maturity level and it shows excellent potential to meet ITRS requirements for next-generation memory technology. Apart from its non-volatility, it shows good scalability potential below 10 nm. Some of the RRAM advantages are summarized in schematic diagram (Figure 1). Ho et al. [28] has demonstrated a 9-nm half-pitch RRAM device. They showed that if high-density vertical bipolar junction transistor will be used as a select transistor, it cannot provide the programming current required for PCRAM below 40 nm while for RRAM, it can be used even below 10 nm. Park et al. [20] reported sub-5-nm device in a Pt/TiO_2_/Cu structure. Ultra-high-speed operation of RRAM using atomic layer deposited HfO_2_ switching material is reported by Lee et al. [29], where a 300-ps pulse of only 1.4 V, successfully switches the device without any change in memory window. Torrezan et al. [21] also demonstrated the fast switching speed of 105 ps. Low energy consumption of only 0.1 pJ per operation [25] and multi-level data storage [16] required for high-density integration were reported. The energy consumption can be further reduced with increased reliability by scaling it to smaller dimensions [30]. Long pulse endurance of >10^12^ cycles is also demonstrated in TaO_*x*_-based crossbar device [31]. Other incentives of RRAM include its simple metal-insulator-metal (MIM) structure and good complementary metal-oxide-semiconductor (CMOS) compatibility. However, the poor understanding of the switching reliability, mechanism, low-current operation (<100 μA) are the bottlenecks in its further development and optimization. Overall, on the light of above discussion, RRAM is one of the most promising candidates for the replacement of flash in future. On the other hand, RRAM can also find its own application area, which will be more challenging and useful in the near future. Furthermore, the TaO_*x*_-based RRAM devices have been also reported extensively in the literature and shown good resistive switching performance. It is expected that this TaO_*x*_-based RRAM device has strong potential for production in near future. However, the TaO_*x*_-based RRAM devices with prospective and challenges have not been reviewed in literature yet.

This topical review investigates the switching mode, mechanism, and performances of the TaO_*x*_-based devices as compared to other RRAMs in literature. Long program/erase endurance and data retention of >85°C with high yield have a greater prospective of TaO_*x*_-based nanoscale RRAM devices; however, lower current (few microampere) operation is very challenging for practical application, which is reviewed in detail here.

### Resistive RAM overview

**Figure 2:**
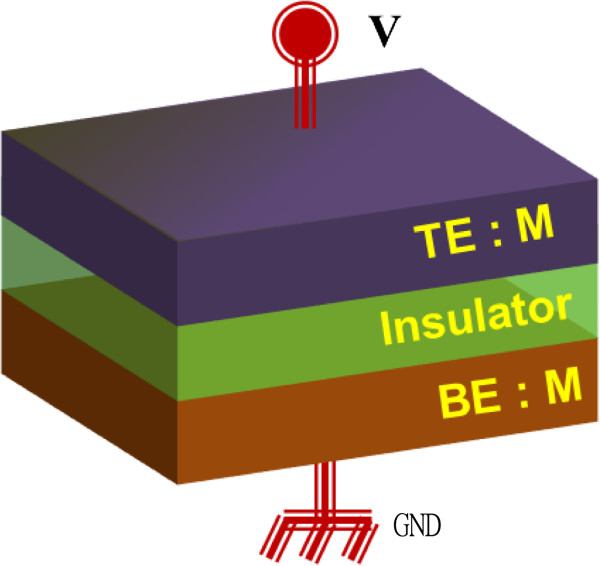
**Structure of RRAM device.** Schematic diagram of RRAM in metal-insulator-metal structure and its biasing.

Resistance switching effect was first reported by Hickmott in 1962 [32] and had subsequently been observed by many researchers over the years [9-36]. RRAM is a two-terminal passive device in which a comparatively insulating switching layer is sandwiched between two electrically conducting electrodes, as shown in Figure 2. However, a working RRAM device generally consists of one transistor (1T) or one diode (1D) and one resistor (1R), i.e., 1T1R or 1D1R configurations. The resistance of the RRAM device can be altered by simply applying external bias across the MIM stack. The electrode on which a voltage or current is applied can be referred to as the top electrode (TE), and the other electrically grounded electrode can be called as the bottom electrode (BE).

#### Switching modes: unipolar/bipolar

**Figure 3:**
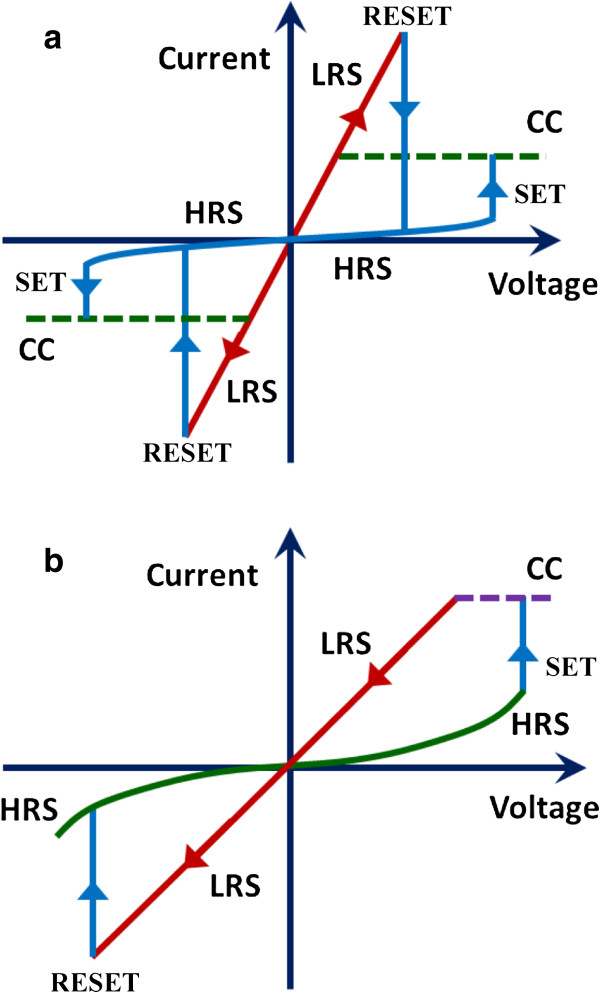
**Switching mode of the RRAM devices. (a)** I-V curves for unipolar (nonpolar) switching where the switching direction is independent on the polarity of the applied voltage and **(b)** bipolar switching. In bipolar switching, SET and RESET occur at opposite polarity bias.

The resistance of a RRAM device can be modulated in two ways as shown by the current/voltage (I-V) curves in Figure 3. On the basis of I-V curves, the switching modes can be classified as unipolar (nonpolar) and bipolar. In unipolar resistive switching mode (Figure 3a), the switching direction does not depend on the polarity of the applied voltage and generally occurs at higher voltage amplitude that of bipolar switching. A pristine memory device with high initial resistance state (IRS) can be switched in to a low-resistance state (LRS) by applying a high voltage stress. This process is called the 'electroforming process’ or simply 'forming process’ and alters the resistance of the pristine device irreversibly [15,37]. Some RRAM devices do not need the forming process and are called forming-free devices. Forming-free devices are highly required for RRAM practical application and are reported infrequently [38-41]. After the forming process, the RRAM device can be switched to a high-resistance state (HRS), generally lower than that of the IRS by the application of a particular voltage called reset voltage. This process is called 'RESET process.’ Switching from a HRS to a LRS called 'SET.’ In the SET process, generally, the current is limited by the current compliance (CC) in order to avoid device damage. The resistive switching in unipolar mode has been observed in many highly insulating oxides, such as binary metal oxides [10]. The unipolar devices suffer from high non-uniformity and poor endurance. In bipolar resistive switching mode, the SET and RESET occur in the opposite polarity, i.e., if memory device can be set by applying positive voltage on TE, then only negative voltage can reset the device (Figure 3b). So, this type of resistive switching is sensitive to the polarity of the applied voltage. For bipolar switching to occur, the MIM stack should be asymmetric generally, such as different electrodes or a dedicated voltage polarity for the forming process. Many oxides show bipolar resistive switching and will be also discussed later. The devices in which unipolar and bipolar modes can be changed by changing the operation conditions are called 'nonpolar’ devices [42], and the resistive switching mechanism is explained below.

#### Resistive switching mechanism

**Figure 4:**
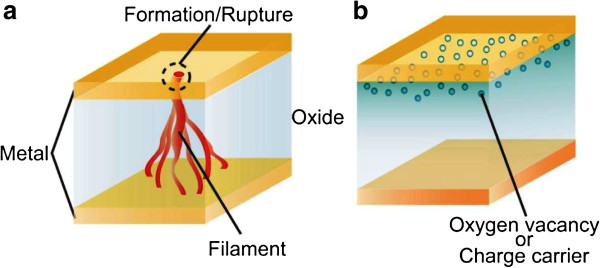
**Switching mechanism. (a)** Filamentary conducting path model and **(b)** an interface-type conducting path model [15,17].

Generally, depending on the conduction path, the switching mechanism can be classified as (1) filamentary-type and (2) interface-type, as shown in Figure 4. In the filamentary model, the switching originates from the formation/rupture of conducting filament in the switching material by the application of suitable external bias shown in Figure 4a [15,17]. The filamentary paths are formed under SET and ruptured under RESET. Electrochemical migration of oxygen ions and redox reaction near the metal/oxide interface is widely considered as the possible mechanism behind the formation and rupture of the filaments [43]. However, clear visualization of the conducting filaments in switching material has yet to be achieved. Studies involving high-resolution transmission electron microscopy showed the conducting filaments in different systems [24,44-48]; however, the switching mechanism is still clearly not understood. On the other hand, in the interface-type mechanism, the switching occurs at the interface of the metal and switching material, as shown in Figure 4b [49]. Several models have been reported for the driving mechanism involved in an interface-type conducting path, such as electrochemical migration of oxygen vacancies [50-53], trapping of charge carriers (hole or electron) [54,55], and a Mott transition induced by carriers doped at the interface [56-58]. To understand the difference between the filament and interface types of resistive switching, the area dependence of the RRAM device resistance could be examined. In general, if the resistance of the LRS is independent of the device area and HRS varies inversely, the switching is filamentary. When both LRS and HRS increase with decreasing device area, the switching is related to interface-type.

Further, depending on the switching material and electrodes, the resistive switching memory can be divided into two types: cation-based switching called electrochemical metallization (ECM) memory and anion-based switching called valance change memory (VCM) [17]. In cation-based memory, a solid-electrolyte was used as a switching material and an electrochemically active metal such as copper (Cu), silver (Ag), and Nickel (Ni) as TE and an inert metal as BE [17]. Generally, the ions of Cu and Ag were known as mobile ions. When positive voltage was applied on the Cu TE, for example, metallic Cu was reduced electrochemically to give Cu^+^ ions generated from metallic Cu due to anodic dissolution. These ions then diffused through the solid electrolyte due to electric field and reached to the BE where these ions reduced to become metallic Cu and electro-crystallize on the BE. As a result, a conducting filament grew preferentially from the BE and finally bridge the BE and TE. Consequently, the device switched to the LRS. That is the reason that ECM devices were also called conducting bridge RAM. When negative voltage was applied on the TE electrode, the Cu filament broken due to electrochemical dissolution reaction initiated by an electronic current through the metallic bridge, and, in parallel, an electrochemical current and the device came into HRS. In recent years, many solid electrolyte materials such as GeSe_*x*_[11,59,60], GeS [61,62], Cu_2_S [63], Ag_2_S [64], Ta_2_O_5_[65,66], SiO_2_[67], TiO_2_[68], ZrO_2_[69], HfO_2_[70], GeO_*x*_[48], MoO_*x*_/GdO_*x*_[71], TiO_*x*_/TaSiO_*y*_[72], GeSe_*x*_/TaO_*x*_[46], CuTe/Al_2_O_3_[73], and Ti/TaO_*x*_[22] were reported. The VCM devices consist of a sub-stoichiometric switching material and an inert electrode such as Pt, Ir, Au, etc., or reactive electrode such as W, Al, Ti, Ni, etc. In VCM devices, switching occurs due to the redox reaction induced by anion (O^2-^) migration to form conducting filament, as shown in Figure 4a. These devices usually need a forming step in order to switch between LRS and HRS reversibly [17,21]. During electroforming process, the generation of oxygen O^2-^ ions occurs in the switching material due to chemical bond breaking. The generated O^2-^ ions migrate toward the TE under the external bias, and oxygen gas evolution at the anode due to anodic reaction are also reported in literature. To maintain the charge neutrality, the valance state of the cations changes. Therefore, it is called VCM memory. Due to O^2-^ ion generation and anodic reaction, oxygen vacancy conducting path generates in the switching material between TE and BE, and device switches to LRS. The electroforming conditions strongly depend on the dimension of the sample, in particular, the switching material thickness. In addition, thermal effects play an essential role in the electroforming, and it sometimes damage the devices by introducing morphological changes [17,21]. Partially blown electrodes during forming have been observed [17]. Thus, the high-voltage forming step needs to be eliminated in order to product the RRAM devices in future. However, anion-based switching material with combination of different electrode materials and interface engineering will have good flexibility to obtain proper RRAM device.

#### RRAM materials

Resistance switching can originate from a variety of defects that alter electronic transport rather than a specific electronic structure of insulating materials, and consequently, almost all insulating oxides exhibit resistance switching behavior. Over the years, several materials in different structures have been reported for RRAM application to have better performance. The switching materials of anion-based devices include transition metal oxides, complex oxides, large bandgap dielectrics, nitrides, and chalcogenides. Table 1 lists some of the important materials known to exhibit resistance switching for prospective applications. Few of them reported low-current operation <100 μA only, which is very challenging for real applications in future. Among other various metal oxides such as NiO_*x*_[74-76], TiO_*x*_[77-81], HfO_*x*_[29,38,82-86], Cu_2_O [87], SrTiO_3_[43,88], ZrO_2_[89-92], WO_*x*_[28,30,93], AlO_*x*_[94-97], ZnO_*x*_[39,98-101], SiO_*x*_[102,103], GdO_*x*_[104,105], Pr_0.7_Ca_0.3_MnO_3_[15,106], GeO_*x*_[107,108], and tantalum oxide (TaO_*x*_)-based devices [31,109-128] are becoming attractive owing to their ease of deposition using existing conventional systems, high thermal stability up to 1,000°C [115], chemical inertness, compatibility with CMOS processes, and high dielectric constant (ϵ = 25). Moreover, Ta-O system has only two stable phases of Ta_2_O_5_ and TaO_2_ with large solubility of O (71.43 to 66.67 at.%) above 1,000°C in its phase diagram [129]. This property of TaO_*x*_ is important in order to achieve long switching endurance (the longest reported endurance of >10^12^ cycles is from TaO_*x*_-based device [31]). It has a metastable and comparatively conducting TaO_2_ phase. Further, the absolute value of Gibbs free energy for redox (reduction-oxidation) reaction of TaO_*x*_ is low which shows its better stability [109]. The redox reaction is written in Equation 1 below.

(1) $$2\mathrm{Ta}O_{2}+O^{2}\Leftrightarrow Ta_{2}O_{5}+2e$$

**Table 1:** Switching materials and SET/RESET current in published literature

**RRAM materials with structure**	**Switching mode**	**Current**		**References**
		**SET**	**RESET**	
Pt/NiO/Pt	Unipolar	1 mA	>1 mA	Kim et al. [74]
Pt/NiO/W	Unipolar	~20 μA	~500 μA	Ielmini et al. [75]
Pt/NiO/Pt	Bipolar	3 mA	~3 mA	Jousseaume et al. [76]
Pt/TiO_2_/TiO_2-*x*_/Pt	Bipolar	<200 μA	<200 μA	Yang et al. [77]
Pt/Ti/TiO_2_/W and Pt/W/TiO_2_/W	Bipolar	500 μA	0.5 and 3 mA	Harmes et al. [78]
Ir/TiO_*x*_/TiN	Bipolar	1 mA	~2 mA	Park et al. [79]
TiN/TiO_*x*_/HfO_*x*_/TiN	Bipolar	40-200 μA	40-200 μA	Lee & Chen et al. [29,38]
Pt/ZrO_*x*_/HfO_*x*_/TiN	Bipolar	<200 μA	~200 μA	Lee et al. [83]
TiN/Ti/HfO_2_/TiN	Bipolar	150 μA	~100 μA	Walczyk et al. [84]
Ta/HfO_2_/TiN	Bipolar	100 μA	--	Chen et al. [85]
TiN/TiON/HfO_*x*_/Pt	Bipolar	50 μA	3050 μA	Yu et al. [86]
Ni or Co/Cu_2_O/Cu	Unipolar	~80 μA	~100 μA	Chen et al. [87]
Au or Pt/SrTiO_3_/Au or Pt	Bipolar	2.8 ± 0.8 mA	2.5 ± 0.5 mA	Szot et al. [43]
Au/SrTiO_3_/Ti	Bipolar	10 mA	~2 mA	Sun et al. [88]
Ti/ZrO_2_/Pt	Bipolar	30 mA (self)	~30 mA	Lin et al. [89]
Cu/ZrO_2_:Ti/Pt	Bipolar	1 mA	~10 mA	Liu et al. [90]
Ti/ZrO_2_/Pt	Bipolar	5 mA	~4 mA	Wang et al. [91]
Ti/Mo:ZrO_2_/Pt	Bipolar	<20 mA	<30 mA	Wang et al. [92]
TiON/WO_*x*_/W/TiN	Bipolar	100 nA	1 μA	Ho et al. [28]
TiN/WO_*x*_/W	Unipolar	--	--	Chien et al. [93]
Pt/WO_*x*_/W	Bipolar	10 mA	~10 mA	Kim et al. [30]
Ti/Al_2_O_3_/Pt	Bipolar	>1 mA	~7 mA	Lin et al. [94]
Pt/Al_2_O_3_/TiN	Bipolar	20 μA	~20 μA	Wu et al. [96]
IrO_*x*_/Al_2_O_3_/IrO_*x*_ND/Al_2_O_3_/IrO_*x*_	Bipolar	500 μA	>1 mA	Banerjee et al. [97]
Cu/ZnO/n^+^	Unipolar	~500 μA	~3 mA	Qinan et al. [39]
Pt/Mn:ZnO/Pt	Unipolar	5 mA	~17 mA	Peng et al. [98]
Ti/ZnO/Ti	Nonpolar	20 mA	--	Andy et al. [99]
Pt/ZnO/Pt	Bipolar	3 mA	~3 mA	Chiu et al. [100]
Au/ZnO/Au	Bipolar	10 mA	~10 mA	Peng et al. [101]
TiW/SiO_*x*_/TiW	Unipolar	~100 μA	~200 μA	Yao et al. [102]
n-Si/SiO_*x*_/p-Si	Bipolar	2 μA	~100 μA	Mehonic et al. [103]
Pt/Gd_2_O_3_/Pt	Unipolar	10 mA	~30 mA	Cao et al. [104]
IrO_*x*_/GdO_*x*_/WO_*x*_/W	Bipolar	1 mA	~1 mA	Jana et al. [105]
Pt/Al/Pr_0.7_Ca_0.3_MnO_3_/Pt	Bipolar	1 mA	~10 μA	Seong et al. [106]
Ni/GeO_*x*_/HfON/TaN	Bipolar	0.1 μA (self)	0.3 nA	Cheng et al. [107]
IrO_*x*_/Al_2_O_3_/GeNWs/SiO_2_/p-Si	Bipolar	20 μA	22 μA	Prakash et al. [108]
Pt/TaO_*x*_/Pt	Bipolar	<170 μA	<170 μA	Wei et al. [109]
IrO_*x*_/TaO_*x*_/WO_*x*_/W	Bipolar	1 mA	627 μA	Prakash et al. [116]
Ta/TaO_*x*_/Pt	Bipolar	100 μA	~100 μA	Yang et al. [110]
Pt/Ta_2_O_5-*x*_/TaO_2-*x*_/Pt	Bipolar	200 μA	~200 μA	Lee et al. [31]

A schematic potential energy curve for TaO_*x*_ is reported by Wei et al. [109]. This implies that both the HRS and the LRS of TaO_*x*_ are stable owing to small difference of Gibbs free energy in between LRS and HRS, and the barrier height between these states is quite high. Due to these benefits of TaO_*x*_ switching material, it is important to design RRAM for real application. That is why this material has been studied in this review below.

### Resistive RAM using TaO_*x*_ material

**Figure 5:**
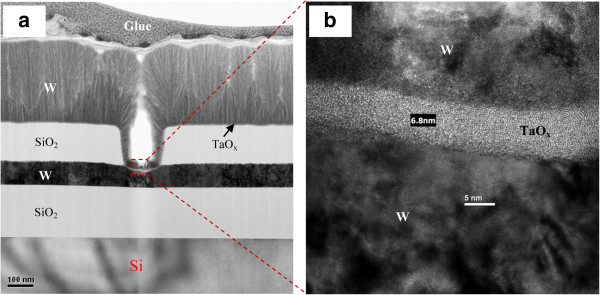
**TEM image of W/TaO**_***x***_**/W structure. (a)** Cross-sectional TEM image with a device size of 0.15 × 0.15 μm^2^. **(b)** HRTEM image inside the via-hole region. The thickness of TaO_*x*_ film is approximately 6.8 nm.

**Figure 6:**
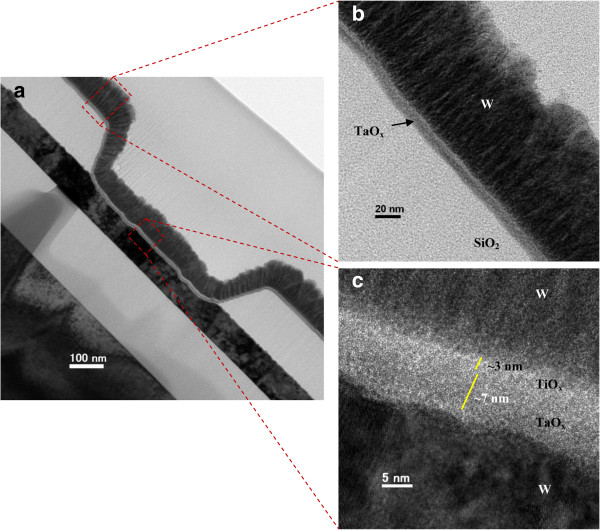
**TEM image of W/TiO**_***x***_**/TaO**_***x***_**/W structure. (a)** Cross-sectional TEM image with a typical device size of 0.6 × 0.6 μm^2^. HRTEM images of **(b)** outside and **(c)** inside via-hole regions.

**Figure 7:**
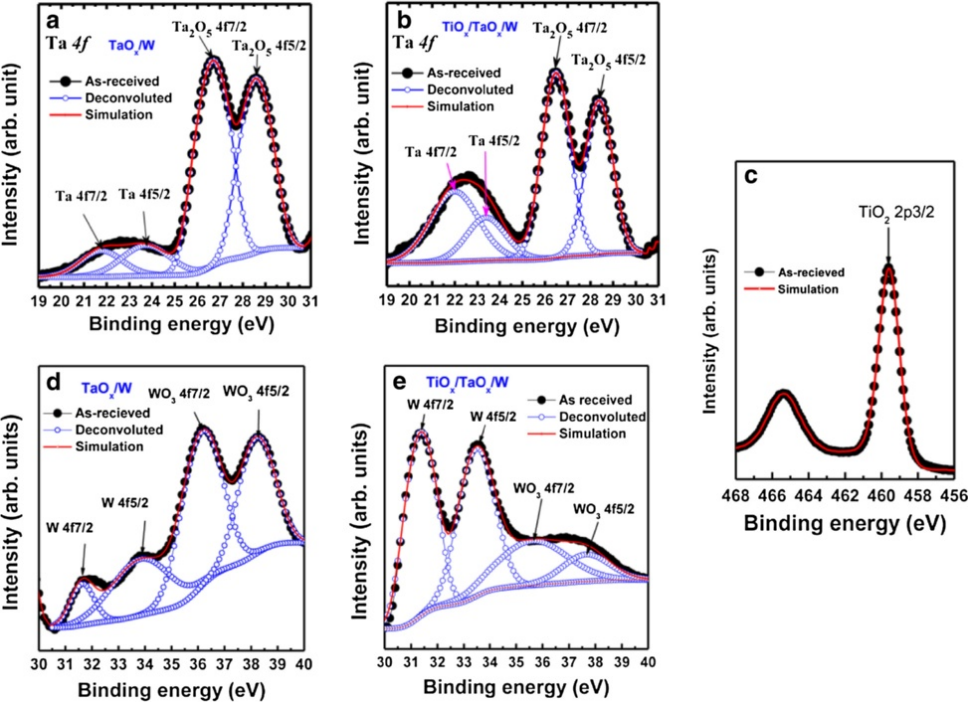
**XPS characteristics.** Ta *4f* spectra for **(a)** TaO_*x*_/W and **(b)** TiO_*x*_/TaO_*x*_/W structures. **(c)** Ti *2p* spectrum. W *4f* and WO_3_*4f* spectra for the **(d)** TaO_*x*_/W and **(e)** TiO_*x*_/TaO_*x*_/W structures [22,114].

A small via size of 150 × 150 nm^2^ of the W/Ti/TaO_*x*_/W and W/TaO_*x*_/W structures was fabricated [41]. A high-κ Ta_2_O_5_ film with a thickness of ≈7 nm was then deposited by an e-beam evaporator. Then, a thin Ti (≈3 nm) interfacial layer by rf sputtering was deposited. The final devices were obtained after a lift-off process. Memory device structure and thicknesses of all layers were observed by transmission electron microscopy (TEM) with an energy of 200 keV. Figure 5a shows a typical cross-sectional TEM image of the W/TaO_*x*_/W structure. The device size is 150 × 150 nm^2^. The thickness of TaO_*x*_ layer is 6.8 nm (Figure 5b). Figure 6a shows TEM image of the W/TiO_*x*_/TaO_*x*_/W structures. The thicknesses of the TiO_*x*_ and TaO_*x*_ layers are approximately 3 and 7 nm, respectively. Both films show an amorphous characteristics outside (Figure 6b) and inside (Figure 6c) regions of the via-hole. The device size is approximately 0.6× 0.6 μm^2^. As Ti removes oxygen from the Ta_2_O_5_ film in the W/TiO_*x*_/TaO_*x*_/W structure, the film becomes more oxygen-deficient TaO_*x*_, which is very important to achieve an improved resistive switching. XPS analyses were carried out to determine the oxidation states of all layers after the fabrication process, and the resulting spectra are presented in Figure 7[22,114]. The spectra were simulated using Gaussian-Lorentzian functions. The peak binding energies of Ta_2_O_5_ 4f_7/2_ and Ta_2_O_5_ 4f_5/2_ electrons for the Ta_2_O_5_/W structure were centered at 26.7 and 28.6 eV, respectively (Figure 7a), and the binding energies of Ta 4f_7/2_ and Ta 4f_5/2_ electrons were centered at 21.77 and 23.74 eV, respectively. This suggests that the high-κ Ta_2_O_5_ film mixed with Ta metal, resulting in a TaO_*x*_ layer where *x*< 2.5. This may be due to the reaction of oxygen with the bottom W layer during deposition of the Ta_2_O_5_ film. It is very interesting to note that the area ratios of the Ta 4f_7/2_ and Ta 4f_5/2_ peaks with respect to the area of the Ta_2_O_5_ 4f_7/2_ peak are both 0.03 for the TaO_*x*_/W structure, while those of the TiO_*x*_/TaO_*x*_/W structure are 0.27 and 0.16, respectively (Figure 7b). This means that the Ta content of the TiO_*x*_/TaO_*x*_/W structure was higher than that of the TaO_*x*_/W structure. Furthermore, the binding energy of TiO_2_ 2p_3/2_ in Ti/TaO_*x*_/W structure is 459.57 eV (Figure 7c). As Ti removes oxygen from the Ta_2_O_5_ film, the film becomes the more oxygen-deficient TaO_*x*_, which is vital to achieve improved resistive switching. The peak binding energies of the W 4f_7/2_, WO_3_ 4f_7/2_, W 4f_5/2_, and WO_3_ 4f_5/2_ electrons of the TaO_*x*_/W structure are centered at 31.6, 36.2, 33.9, and 38.3 eV, respectively (Figure 7d). The area ratios of the WO_3_ 4f_7/2_ and WO_3_4f_5/2_ spectra with respect to the area of W 4f_7/2_ are both 0.03 for the TaO_*x*_/W structure, while those for the TiO_*x*_/TaO_*x*_/W structure are 0.27 and 0.16, respectively (Figure 7e). This suggests that W can be oxidized at the TaO_*x*_/W interface when a Ti layer is not present, resulting in a TaO_*x*_/WO_*x*_/W structure which may have inferior resistive switching properties. When a Ti layer is deposited on the TaO_*x*_ film, the W layer is prevented from oxidizing at the TaO_*x*_/W interface, leading to the formation of a TiO_*x*_/TaO_*x*_/W structure. Considering the Gibbs free energies of TiO_2_, Ta_2_O_5_, and WO_3_ films, which are -887.6, –760.5, and -506.5 kJ/mol, respectively, at 300 K [130], the Ti will consume the highest oxygen content owing to its stronger reactivity than those of the other materials, thereby forming Ta-rich (or defective TaO_*x*_) film. This also prevents oxidation of the W TE at the TaO_*x*_/W interface owing to the migration of oxygen from the underlying films toward the Ti film, which contributes to the improved resistive switching memory performance as described below.

**Figure 8:**
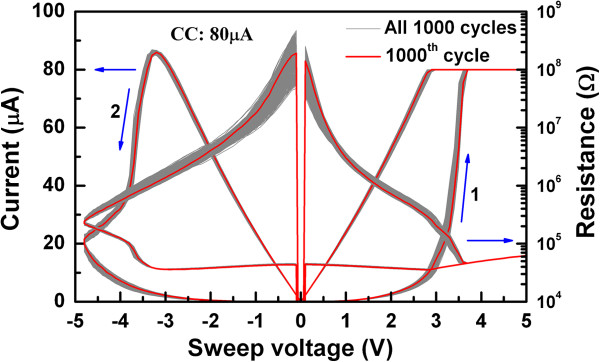
**Switching characteristics.** Consecutive 1,000 current/voltage and resistance-voltage characteristics of Ti interfacial layer in the W/TiO_*x*_/TaO_*x*_/W devices [41].

Resistive switching memory characteristics are explained here. Figure 8 shows current/voltage and resistance-voltage characteristics. The W/TiO_*x*_/TaO_*x*_/W device exhibits >1,000 consecutive repeatable dc switching cycles with a better resistance ratio of 10^2^ under a low CC of 80 μA, the W/TaO_*x*_/W device shows few switching cycles with a higher CC of 300 μA [41]. In this case, negatively charged oxygen ions (O^2-^) migrate from the switching material toward W TE, and this has a lesser possibility to form an oxygen-rich layer at the W TE/TaO_*x*_ interface, leading to the formation of multi-conduction filaments. However, the insertion of a thin (≈3 nm) Ti layer in between the W and TaO_*x*_ layers in the W/TiO_*x*_/TaO_*x*_/W device makes a vast difference because Ti can be used as an oxygen reservoir. A repeatable switching of >10,000 cycles is also observed [41]. Under 'SET,’ O^2-^ rather than oxygen vacancies will migrate from TaO_*x*_ toward the TE, resulting in a TiO_2_ layer which controls the conducting vacancy filament diameter in the TaO_*x*_ layer by controlling current overflow and producing a tighter distribution of the LRS. Owing to this series resistance, the devices exhibit non-ohmic current. It is true that the conducting filament is formed through the TaO_*x*_ film. When negative voltage is applied to the TE, oxygen ions are pushed from the TiO_2_ layer toward the conducting filament where they recombine with oxygen vacancies or oxidize the conducting filament. The device will be in HRS. Control of oxygen-deficient filament formation and rupture is facilitated by insertion of the thin Ti layer at the TE/TaO_*x*_ interface, which results in repeatable and reproducible resistive switching characteristics, which has very good prospective of TaO_*x*_-based resistive switching memory in a W/TiO_*x*_/TaO_*x*_/W structure for real application. Some other reported results have been explained below.

**Figure 9:**
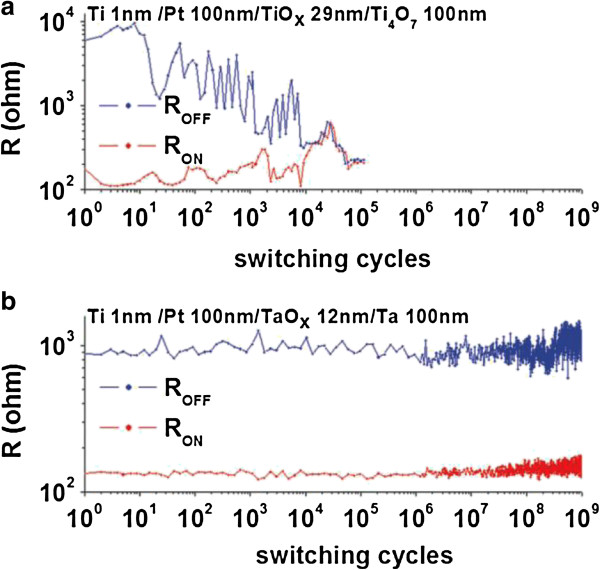
**Program/erase endurance.** Endurance comparison of **(a)** TiO_*x*_ and **(b)** TaO_*x*_ devices [110].

**Figure 10:**
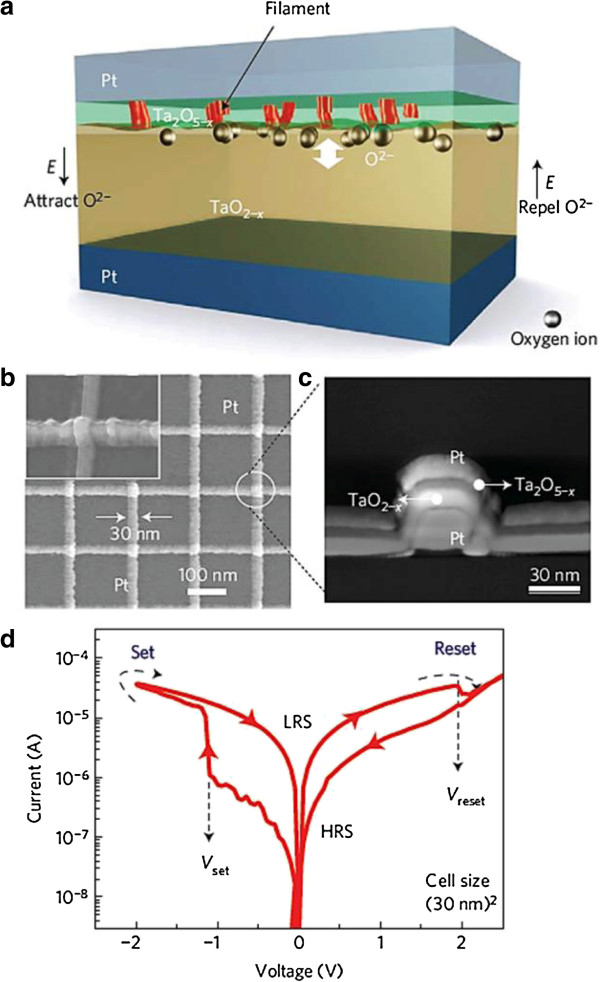
**Schematic of switching mechanism and I-V characteristics of cross-point memory devices. (a)** Schematic representation of the TaO_*x*_ device consisting of a thin Ta_2_O_5-*x*_ insulating layer and a TaO_2-*x*_ base layer. The movement of internal oxygen ions or vacancies is used to model the switching. **(b)** SEM image of a 30-nm crossbar array of devices with the inset showing a single device. **(c)** TEM cross-section of a 30-nm crossbar cell. The total thickness of TaO_*x*_ layer is 30 nm. **(d)** I-V hysteresis characteristics [31].

Yang et al. [110] has reported the Pt/TaO_*x*_/Ta device with a diameter of 100 μm, where Pt was grounded and external bias was on the Ta electrode. Long program/erase (P/E) endurance of 1.5 × 10^10^ cycles with a pulse width of 1 μs is reported. Further, a comparison of endurance characteristics made between TiO_*x*_ and TaO_*x*_-based devices (Figure 9) shows far better performance by TaO_*x*_-based devices stretching the P/E cycles to >10^9^ cycles (Figure 9b) as compared to only 10^4^ cycles for TiO_*x*_-based devices and it is collapsed finally (Figure 9a). The reason having longer endurance in TaO_*x*_ devices is the presence of only two solid stable phases in bulk equilibrium with each other and large oxygen solubility in Ta-O system which can act as the source/sink of mobile ions for switching in the insulating phase as compared to many Magneli phases in Ti-O system [110]. The operation current could be reduced to 100 μA. The underlying switching mechanism is attributed to the redox reaction resulting insulating Ta_2_O_5_ and conducting Ta(O) solid solution. The energy-filtered TEM (EFTEM) zero-loss images and oxygen map of the switching region confirm also the reduction of TaO_*x*_ thickness by half in the active region, and the oxygen content in the reduced region is found as low as that in the Ta electrode. The switching phenomenon is believed to be due to oxygen vacancies and ions through nano-ionic transport and a redox process, and this can be called VCM [17]. A schematic diagram was shown in Figure 10a [31,41,43,131-133]. As suggested previously, an intrinsic Schottky barrier exists between the Pt TE and the Ta_2_O_5-*x*_ layer contact while in the insulating state, and an ohmic contact is formed in the LRS. This suggests that oxygen ion movement under external bias leads to the LRS to HRS or HRS to LRS. Lee et al. [31] reported TaO_*x*_-based crossbar resistive switching memory device. Figure 10b shows the scanning electron microscopy (SEM) image. The device stack consists of Pt top and bottom electrode and bilayer TaO_*x*_ switching layer with insulating Ta_2_O_5-*x*_ layer near TE and TaO_2-*x*_ near BE as can be seen in the cross-section TEM image presented in Figure 10c. They fabricated the devices with different sizes from 50 × 50 μm^2^ to 30 × 30 nm^2^. All size devices have shown self-compliance bipolar switching with small set/rest voltage of -1.0/2.0 V. The switching current of 50 × 50 μm^2^ device was >200 μA and for 30 × 30 nm^2^ device was approximately 40 μA, respectively (Figure 10d). From the I-V switching curves, this is a symmetric current profile when the device is in the LRS, but it is an asymmetric current profile for the HRS. This property was exploited to realize RRAM devices in crossbar architecture without any selection device with anti-serial connection. They were also able to achieve the highest ever reported endurance value of 10^12^ for this system at 30 × 30 μm^2^ cell size for base layer oxidation of 3%. Data retention of >10 years at 85°C was also reported. To eliminate the need for a discrete switch element such as a diode or transistor, they connected two Pt/Ta_2_O_5-*x*_/TaO_2-*x*_/Pt cells antiserially by external contacts and this concept was also reported by Linn et al. [134].

**Figure 11:**
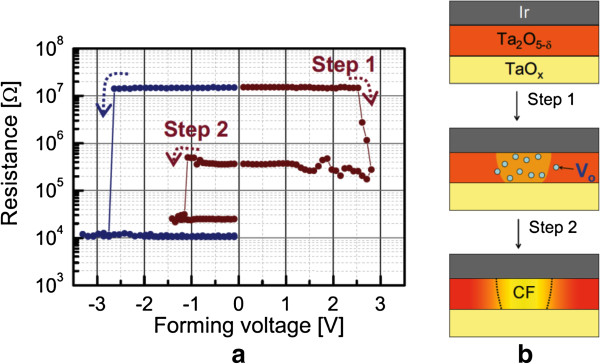
**Electroforming process and filament diameter control. (a)** Pulsed resistance-voltage curve of the two-step forming scheme (red) compared with the common forming scheme (blue). Small conducting filament formation is confirmed by its high resistance after step 2. **(b)** Schematics of the Ta_2_O_5-*δ*_ resistive switching layer during the two-step forming process. Oxygen vacancies are generated in the Ta_2_O_5-*δ*_ layer after step 1, and a conducting filament is formed by applying a negative pulse in step 2 [120].

**Figure 12:**
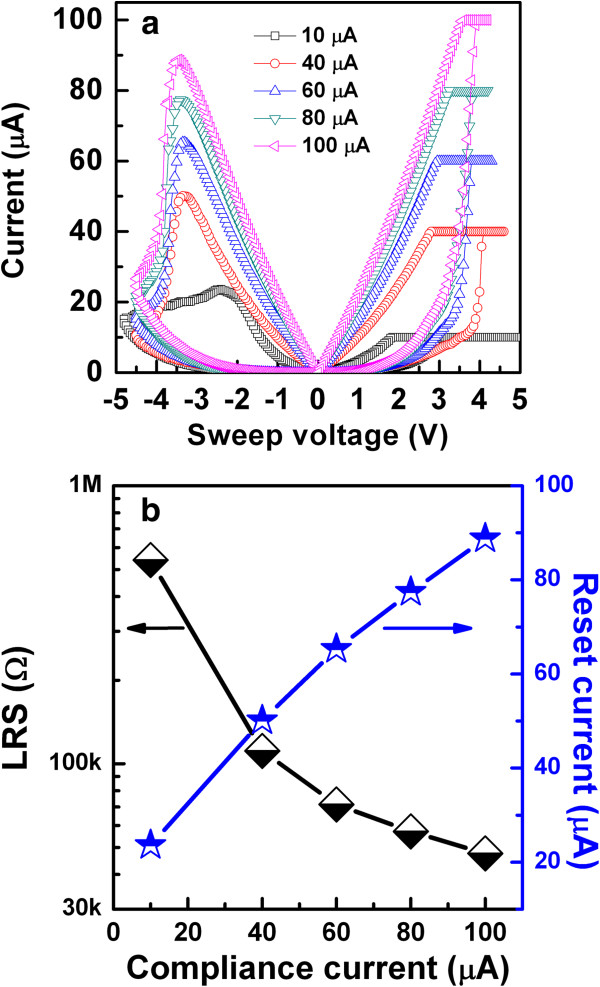
**Current/voltage hysteresis with different current compliances.** I-V hysteresis characteristics **(a)** LRS and reset currents **(b)** with 10- to 100-μA CCs. A device could be operated with a low reset current of 23 μA [41].

**Figure 13:**
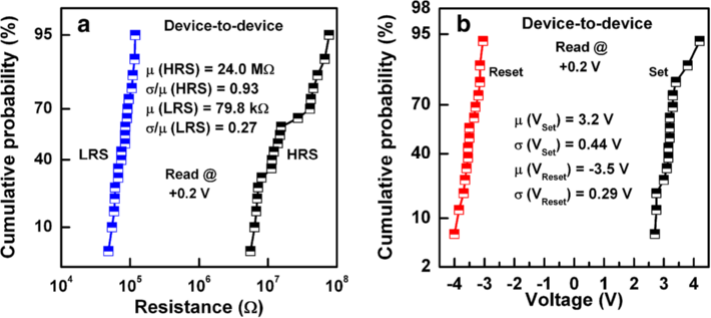
**Statistical data plot.** Cumulative probability plots of **(a)** LRS and HRS and **(b)** SET and RESET voltage.

**Figure 14:**
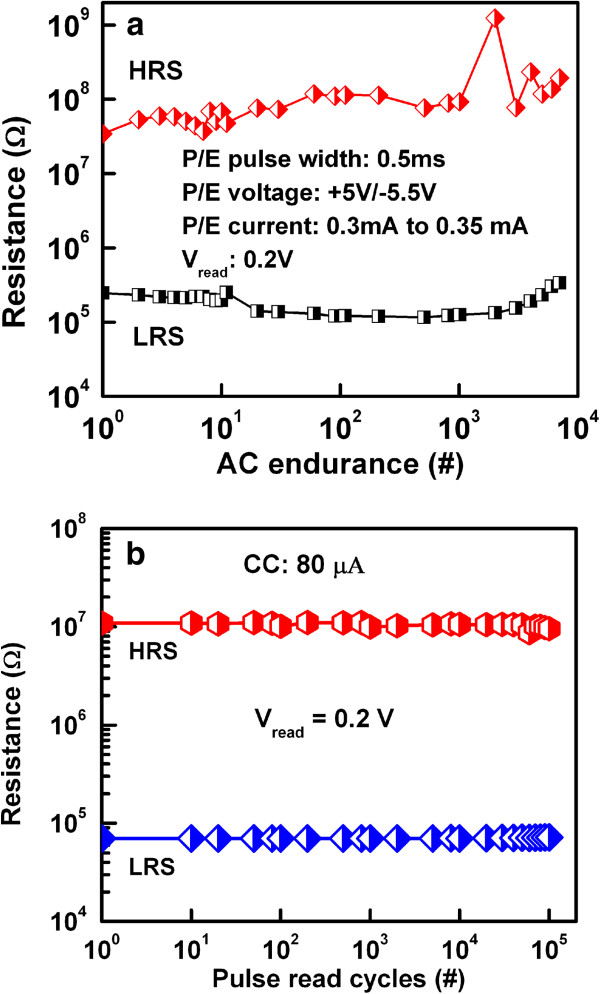
**Endurance characteristics. (a)** AC endurance of >10^4^ cycles and **(b)** long read pulse endurance of >10^5^ cycles at a read voltage of 0.2 V.

**Figure 15:**
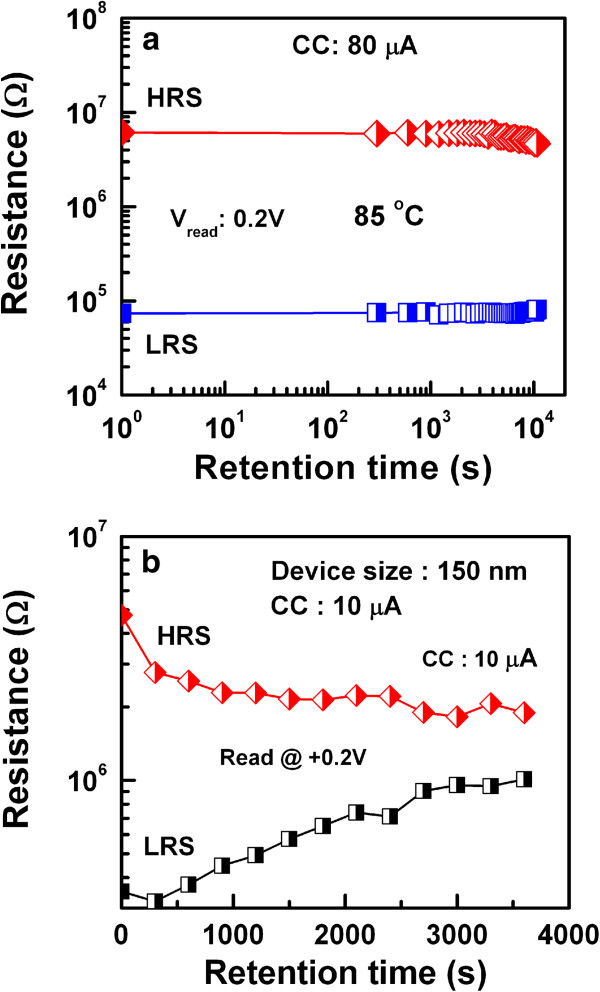
**Data retention characteristics. (a)** Good data retention of >10^4^ s with a good resistance ratio of >10^2^ at 85°C under CC of 80 μA and **(b)** the resistance ratio gradually decreases with retention time at a low CC of 10 μA.

Wei et al. [109] explored first the prospective application of TaO_*x*_-based RRAM devices. The memory stack consisted of Pt top and bottom electrodes and a non-stoichiometric switching layer of TaO_*x*_. The first layer near the TE is close to insulating Ta_2_O_5-*x*_ phase, while the other layer is close to TaO_2-*x*_ phase which is conducting. The memory device with a size of 0.5 × 0.5 μm^2^ in 1T1R configuration showed bipolar switching characteristics under an operation current of approximately 170 μA. The device shows excellent P/E endurance of >10^9^ cycles. The data retention property could be improved under low-current operation by controlling the size of the conductive filament as well as percolation paths, while the density of oxygen vacancy is kept high enough. It is true that the conducting filament size can be scaled down by reducing both the forming current and formation. A forming voltage can be decreased with a thinner switching layer. However, the thinnest layer is not required because this will have lower HRS. Figure 11a shows a pulsed R-V curve of the two-step forming to control the formation of conducting filament size in Ir/Ta_2_O_5-*δ*_/TaO_*x*_ resistive memory stack [120]. At first (or step 1), a positive pulse that has the same polarity for the RESET is applied to generate oxygen vacancies in the Ta_2_O_5-*δ*_ layer, as shown in Figure 11b. The resistance of the switching material decreases drastically from the initial resistance state (IRS: approximately 15 MΩ); however, it stays at HRS (200 to 500 kΩ), as shown in red line. Second (or step 2), a negative pulse is applied to create the conducting filament at LRS (approximately 20 kΩ). A negative forming voltage, which determines the conducting filament size, is reduced from 2.6 to 1.1 V with a 100-ns pulse width. However, a conventional negative forming voltage (-2.6 V) is shown in blue line, this changes HRS (approximately 15 MΩ) to LRS (approximately 10 kΩ). Quantum-size effect and percolation models of RESET for different switching materials have been explained to understand the conducting filaments [135,136]. Another method of reducing CC can be used to control the conducting filament size, which can be achieved by adjusting the resistivity of the bulk TaO_*x*_ layer. The resistivity can reduce the forming current by controlling the oxygen content of TaO_*x*_[120]. In this case, the conducting filament size becomes smaller and oxygen vacancy becomes larger when the oxygen content is increased. The observed switching is due to the change of barrier height on the application of voltage. When positive voltage was applied, O^2-^ ions migrate from bulk and accumulate near the TE. Oxidation reaction increases the barrier height and device comes to the HRS. On the other hand, when negative voltage was applied on the TE, O^2-^ ions move away from TE and reduction reaction lowers the barrier height which brings the device into LRS. Hence, the barrier height change on the application of bias voltage due to redox reaction is responsible for the observed switching. Several kinds of electrode materials were examined and found that the materials having high work function show stable resistance switching behavior. The significant improvement in the retention characteristics at 150°C under the small current operation of 80 μA by two-step forming are obtained as compared to single-step forming. Two-step electroforming process is very critical to have controlled conducting filament diameter as well as the RRAM could be operated as low current at 80 μA. The W/TiO_*x*_/TaO_*x*_/W memory device showed good bipolar resistive switching characteristics with different CCs from 10 to 100 μA (Figure 12[41]). The low-resistance state decreases with increasing CCs from 10 to 100 μA (Figure 12a,b), which will be useful for multi-level data storage applications. As the filament diameter increases with higher CCs, the low-resistance state decreases, and the value of RESET voltage increases. The RESET current can be scaled down to 23 μA at a low CC of 10 μA. Figure 13a,b shows the device-to-device uniformity of LRS/HRS and SET/RESET voltage, respectively. The cumulative probability distribution is small for both LRS/HRS as well as set/reset voltage. The resistance ratio of HRS/LRS is >100, and the device can be operated below ±5 V. The device can be switched more than 10^4^ AC cycles with stable LRS, as shown in Figure 14a. The device has also shown good read endurance of >10^5^ times at a read voltage of 0.2 V (Figure 14b). No read disturbance is observed during whole course of testing. Figure 15a shows the data retention characteristics at high temperature of 85°C under small switching current of 80 μA. Good data retention of both the states is obtained for >10^4^ s with memory margin of >10^2^. Considering the obtained nano-filament diameter of approximately 3 nm [41], a high density of approximately 100 Tbit/in^2^ is obtained. This device has shown also data retention of few minutes at a very low current of only 10 μA, as shown in Figure 15b. The resistance ratio is gradually decreased with elapsed time. Table 2 compares data published in literature for TaO_*x*_-based resistive switching memories [16,31,41,83,85,109,120] and other materials [137-140]. It is found that TaO_*x*_-based resistive switching devices is one of the comparative materials with other switching materials; however, the low-current operation is published a few papers. This suggests that the TaO_*x*_-based RRAM devices with low-current operation are a big challenging for real application, which needs to be studied in future.

**Table 2:** Data comparison in published literature

**Device structure**	**Device size (μm**^**2**^**)**	**Set/reset voltage (V)**	**Current compliance (μA)**	**Retention (s)**	**Resistance ratio**	**Endurance (cycles)**
W/TiO_*x*_/TaO_*x*_/TiN [41]	0.15 × 0.15	3.0/-3.0	80	>3 h, 85°C	100	10^4^
Ir or Pt/Ta_2_O_5-*δ*_Ta_2-*β*_/Pt [109,120]	0.5 × 0.5	-1/+0.8	80/150	>10^7^	~10	10^9^
Pt/Ta_2_O_5-*x*_/TaO_2-*x*_/Pt [31]	50 × 50-0.03 × 0.03	-2.0/+2.0	40-200	10 years, 85°C	~10	10^12^
Ru/Ta_2_O_5_/TiO_2_/Ru [137]	4 × 4	+2.7/-1.0	~100	>10^6^	~50	10^6^
TiN/Ti/HfO_*x*_/TiN [16,138]	~0.4 × 0.4-0.03 × 0.03	1.0/-1.5	40, 200	>10^4^, 200°C	~100	10^8^
Hf, Ti, Ta/HfO_2_/TiN [85]	0.04 × 0.04	+1.8/-3	100	>10^4^, 200°C	~10	10^10^
TiN/Hf/HfO_2_/TiN [139]	0.01 × 0.01	±0.5	<80	10^5^, 200°C	~100	5 × 10^7^
Pt/ZrO_*x*_/HfO_*x*_/TiN [83]	0.05 × 0.05	0.6/-1.5	50	10^5^, 125°C	~100	10^6^
TiN/WO_*x*_/TiN [140]	0.06 × 0.06	-1.4/+1.6	400	2 × 10^3^ h, 150°C	~10	10^6^

## Conclusions

It is reviewed that TaO_*x*_-based bipolar resistive switching memory could be operated at a low current of 80 μA [41,109], which has prospective of RRAM applications in the future. Further, TaO_*x*_ is a simple and useful material because of two stable phases of TaO_2_ and Ta_2_O_5_, as compared to other reported materials. Long program/erase endurance of >10^10^ and 10 years data retention are also reported in published literature [31,110]. So far, bilayered TaO_*x*_ with inert electrodes (Pt and/or Ir) or single-layer TaO_*x*_ with semi-reactive electrodes (W and Ti/W or Ta/Pt) are reported; however, conducting nano-filament formation/rupture is controlled by oxygen ion migration through bilayered or interfacial layer design under external bias. Further, high-density memory with a small size of 30 × 30 nm^2^ could be designed using crossbar architecture [31]. It is found that the memory performance is becoming worst at operation current of 10 μA. Therefore, it is very challenging to reduce the operation current (few microampere) of the RRAM devices. So far, good performance of TaO_*x*_-based resistive switching memory devices is investigated, as compared to other switching materials in different RRAMs. This topical review shows good prospective; however, it needs to overcome the challenges for future production of the TaO_*x*_-based nanoscale RRAM application.

## Competing interests

The authors declare that they have no competing interests.

## Authors’ contributions

AP and DJ reviewed the papers under the instruction of SM. AP wrote the first draft and DJ prepared Tables 1 and 2 carefully under the instruction of SM. The final draft was modified by SM. All authors read and approved the final manuscript.
